# Clogged information flow and stock-market sluggishness

**DOI:** 10.1371/journal.pone.0235978

**Published:** 2020-07-14

**Authors:** Premal P. Vora

**Affiliations:** School of Business Administration, Penn State Harrisburg, Middletown, PA, United States of America; The Bucharest University of Economic Studies, ROMANIA

## Abstract

I provide evidence on the trading effects of the 1970 Newspaper and Mail Deliverers’ Union’s strike against *The Wall Street Journal*. I find that turnover falls significantly on the first few days of the strike, returns to normal, then even exceeds the average as the strike proceeds. The evidence is consistent with the idea that a clogged information flow causes sluggishness in the market but consumers of media substitute one source for another when their preferred source is clogged. The effects are widespread without regard to firm size or other firm characteristics. When information is clogged, I find that return comovement among assets increases. Finally, I also find that turnover is closely related to the publication of an article in the *Journal* and to positive and negative abnormal returns, but responds more to the latter.

## Introduction

One of the longest-lasting and most significant paradigms of the financial market is that of market efficiency. Asset prices in an efficient market reflect their fundamental value, thereby promoting the efficient allocation of resources in the economy. However, there appear to be many departures from this idyllic view of the market, all well-documented in the literature (see [1]) for a list of the top 11 well-documented departures from market efficiency). It is less well known how these departures occur and why—once found—are not arbitraged away.

There are several good candidate explanations for why departures from market efficiency occur and are not arbitraged away. First, it is possible that investors have behavioral biases that lead to departures from efficiency [2] and those biases continue to sustain inefficiencies. Second, in the presence of noise traders it is possible that the market departs from efficiency and these very traders bring about noise-trader risk that acts to limit arbitrage by arbitrageurs [3].

Third—and most relevant to this paper—is the possibility that investors do not have all the necessary information to make rational decisions about pricing. Even in the absence of behavioral biases and noise traders, if investors do not have all necessary information about the structural parameters of the economy, departures from market efficiency may occur [4]. Investors may not have all the necessary information because information is costly to acquire or the process of information dissemination is slow [5].

In this paper I provide new evidence on what happens to trading activity and market efficiency if the free flow of news or information is somehow clogged. Engelberg and Parsons [6] and Peress [7] examine the effects of clogged news on the stock market, the former by studying the effect of weather-related disruption of newspaper deliveries and the latter by studying newspaper strikes. Both demonstrate that when news is clogged, trading in the stock market falls significantly. Although they do not study the effect of the fall in trading on market efficiency, if trading is the mechanism through which efficiency is restored, the fall in trading could potentially lead to market inefficiencies. I study the Newspaper and Mail Deliverers’ Union strike against *The Wall Street Journal* and its effect on trading on NYSE-listed stocks. Based on newspaper accounst of the strike that appear in “Drivers Strike” [8] and in Robinson [9], the dispute between The Wall Street Journal (WSJ) and the Union stemmed from a new printing facility that WSJ had built in S. Brunswick, NJ, and WSJ’s intention to use labor affiliated with another union to deliver copies from that location. The Union claimed that its members continued to have the right to deliver copies printed at that location. During the eight days of the strike, approximately 100,000 copies of WSJ that were slated for delivery in the New York metropolitan area were not delivered. WSJ was, at the time of the strike and for several decades before and after, widely considered to be the most important source of business and economic news [10]. In 1970, it had a subscription base of greater than 1.2 million copies—the largest of any national daily newspaper. According to the Ayer Directory [11] which covers subscription data for newspapers, in 1970 the subscription numbers were: WSJ: 1,215,750, New York Times: 846,132, Washington Post: 500,118, and LA Times: 966,293. The average daily sales of the NY Daily News, whose focus is on sensational news, was greater than 2 million copies, but many of those were sold at the stand and mostly confined to the NY-NJ metropolitan area. What possible impact could the non-delivery of 100,000 copies out of a subscription base of more than 1.2 million have on trading? That is an empirical question, and as I demonstrate in this paper, it did have a substantial impact on trading.

What is the relevance of research on the effects of a disruption of the delivery of a newspaper in the 1970s to a world where a massive transformation has occurred in how news is generated, disseminated, and consumed? Traditional news gathering organizations play a more vital role today than they did in the past. Due to the reduction in entry barriers to news gathering and dissemination, the need for reputation to build a profitable business has diminished. As a consequence, many news outlets do not check facts before publishing them, nor do they exercise sound editorial judgement [12]. This, coupled with the rapid dissemination of news that occurrs due to technology that is designed to share it, may result in outcomes that are welfare-reducing. For instance, Allcott and Gentzkow [12] study the impact of fake news in the 2016 election and conclude that fake news did have an impact, albeit small, on the outcome. However, this occurred in the presence of an unrestricted flow of news from traditional media outlets. What if, due to some exogenous events, traditional media had been clogged? A stoppage/restriction in the flow of information could occur if vital infrastructure is maliciously hacked.

Schulhofer-Wohl and Garrido [13] study the effect of a closure of a local newspaper in the Cincinnati, Ohio area and find significant changes in the political outcomes in the region. Closer to the world of finance is the finding in Gao, et al. [14] that when local newspapers close, municipal borrowing costs increase significantly. They attribute this effect to the monitoring role that local newspapers play in holding their governments accountable. Thus, it is important to understand the effect that clogging of traditional media has on the market and on social-welfare in general. Additionally, while print media outlets like WSJ were the primary providers of news in the 1970s, there existed alternate channels like radio, television, and wire services. In particular, Dow Jones & Co., the parent of WSJ, owned and operated the Dow Jones News Service (also referred to as the “broadtape”) that disseminated breaking news by wire to its subscribers. According to Rosenberg [15], Dow Jones’ wire service had twice as many subscribers in mid-1970 as its next-closest rival, Reuters. The disruption in the delivery of WSJ due to the strike had no impact whatsoever on wire service. In some ways then, the evidence presented here can be viewed as early evidence on the interplay between print and electronic media and the impact of the disruption of print on the market.

I find that turnover of NYSE-listed firms falls significantly in the first two days of the strike. It rises on the fourth, then it is uneven for the rest of the days. Cross-sectionally, turnover is closely related to the publication of a WSJ article and to residuals from a market model regression—both within and outside the strike period. Further, I find that dispersion ((daily high price—low price)/high price) increases during the strike although it is unclear whether the rise in dispersion is related solely to the occurrence of the strike. I also find that return comovement is higher during the strike than either before or after the strike. As I will explain below, the increase in return comovement is evidence of a decrease in market efficiency. That a clogging of news leads to less efficiency in the market should come as no surprise. However, this research is the first that documents a relation between less news and less efficiency in the market. Additionally, I discover that the general tenor of the news during the strike was negative because returns are consistently negative during the strike. Finally, during 1970 it appears that the firm-specific news that was published in WSJ was negative.

**Fig 1:**
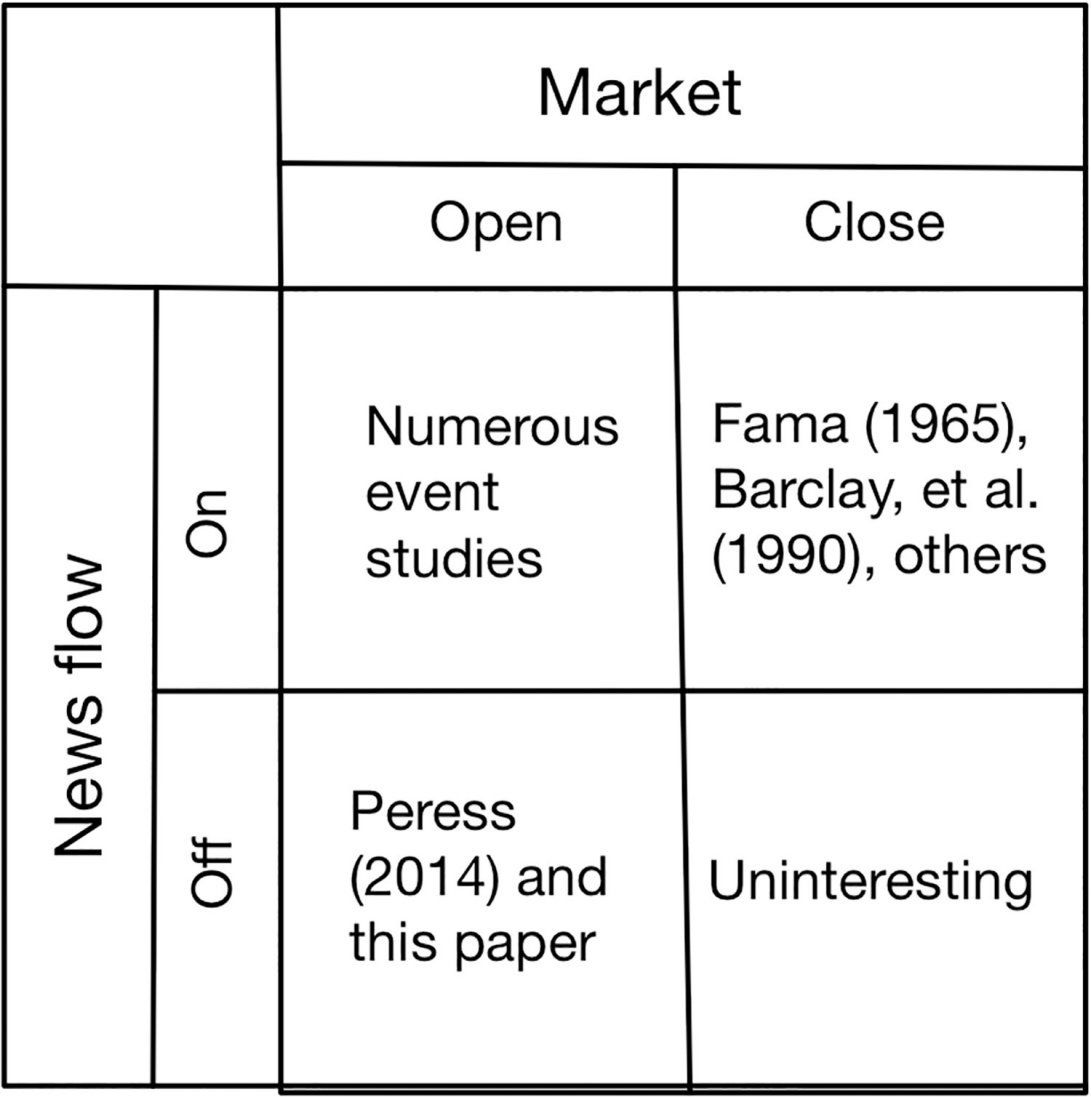
Gap filled by this paper. The financial market can be open or closed. The free flow of news can be on or off. This creates four possible scenarios between the financial market and news flow. This figure captures the gap in the literature that this paper fills.

This paper is related to several research themes in accounting, economics and finance. In Fig 1, I display a 2 × 2 matrix which communicates—visually—the gap in the literature that this paper fills. The financial market can be characterized as being *open* or *closed* and the flow of news can be characterized as being *on* or *off*. That leads to the four permutations—displayed in the matrix—between the state of the financial market and the flow of news. Numerous event studies along the lines of Fama, et al.’s [16] study have been published when the flow of news is on and the financial market is open. Additionally, Fama [17], Barclay, et al. [18], and others have studied stock returns and variances when the market is closed but the flow of news is on. Generally speaking, financial economists have nothing to study when the market is closed and the flow of news is off. The fourth permutation, where the market is open but the flow of news is off, is the one that has hardly received any attention in the literature. In fact, Peress [7] and Engelberg and Parsons [6] are the only studies that I am aware of that examine that situation. Peress’ research is based on events and markets in Europe, and Engelberg and Parsons study the non-delivery of newspapers due to weather in a particular area in a particular state in the US. This paper pertains to an event in the US and the whole US stock market. Thus, it contributes to that significant gap in the literature.

Further, the fall in turnover driven by the clogging of information has implications for how it can affect market efficiency. In a rational expectations equilibrium framework, Veldkamp [19] demonstrates that return comovement between assets increases if information decreases. I provide solid evidence to support this proposition. As Huang, et al. [20] explain, exogenous shocks that reduce investor attention to news leads to higher return comovement between stocks because within the reduced attention investors pay more attention to aggregate news and less attention to firm-specific news. In this paper an exogenous shock reduces the flow of news but the result is identical. In both cases, the increase in return comovement can be interpreted as a decrease in market efficiency.

Additionally, it is related to the literature on the dissemination of information and its *trading* effects on the financial market (see, for instance, [21, 22, 23, 24, 6, 25, 7, 26]). The idea that new information is related to increased trading activity in the market and changes in prices is well established. However, the *causal* relation between media and the financial markets is only tentative. This is because it is possible that the two phenomena are jointly dependent on each other. Both Engelberg and Parsons [6] and Peress [7] get around this difficulty by studying an exogenous disturbance in the flow of news. In particular, Peress focuses on newspaper strikes in countries where they occur most frequently: France, Greece, Italy, and Norway. In the US, however, newspaper strikes are uncommon. However, toward the end of WWII and particularly right after the war had ended, there were massive strikes in the US across all the major industries including auto, steel, mining, trucking, railroad, and even the first airline strike [27]. During that time, The Newspaper and Mail Deliverers’ Union also struck against newspapers including WSJ; however, because that strike was part of a massive labor movement that was just unshackled from the constraints imposed by the War Labor Board it is impossible to separate the effects of clogging of information flow from other strike-related effects. Thus, I expand the universe of newspaper strikes that are studied by focusing on this particular 1970 strike, but more importantly I provide evidence that even a 10% reduction in the flow of important news can affect trading, that too on the world’s largest and most liquid equity market. Finally, it also adds to the literature on trading and its determinants [28, 29].

In the next section I elaborate on the data and the summary statistics. In the following section I present my main findings. I conclude in the last section.

## Data and summary statistics

From CRSP, I gather a list of all common stocks—1,265 altogether—that were listed on the NYSE any time during 1970. I am also able to gather data on daily volume, shares outstanding, the daily high and low price, and daily return for each stock. For each stock and each day, I compute share turnover and dispersion. From the Wall Street Journal Index, I identify the date on which any article pertaining to any of these stocks appeared in WSJ in 1970. There is a large literature in accounting and finance that studies the impact of dividend and earnings announcements on trading. The focus in this paper, however, is on newspaper articles unrelated to dividend and earnings announcements. There are 15,634 such articles in 1970. From Dun & Bradstreet’s Million Dollar Directory [30] I was also able to gather the headquarters address of 1,234 of these firms. Dun & Bradstreet publishes location data on only US-based firms—the missing firms were Canadian or based in Puerto Rico. In Table 1, I display summary statistics on WSJ coverage, the two-digit SIC codes of the sample stocks, the market value of equity, daily turnover and how distant firm headquarters were from WSJ’s headquarters.

**Table 1:** Summary statistics on sample of WSJ articles in 1970 as well as NYSE-listed firms that existed in 1970.

	Min	Mean	Std.	Median	Max	*N*
Number of WSJ articles per stock	0	12.3	20.8	7	338	15,634
Number of WSJ articles per day	25	60.9	12.1	59	108	15,634
Number of firms in two-digit SIC code	1	21.1	25.9	11.5	127	60
Number of WSJ articles in two-digit						
SIC industry/number of firms in industry	2.33	11.02	6.93	9.33	38.58	60
Market value of equity ($ millions)	7.0	469.4	1,743.6	145	41,450.2	1,265
Daily turnover	0.000581	0.001074	0.000284	0.001021	0.002135	254
Distance to WSJ HQ (miles)	0	1,093	1,292.8	718	7,989	1,234

**Fig 2:**
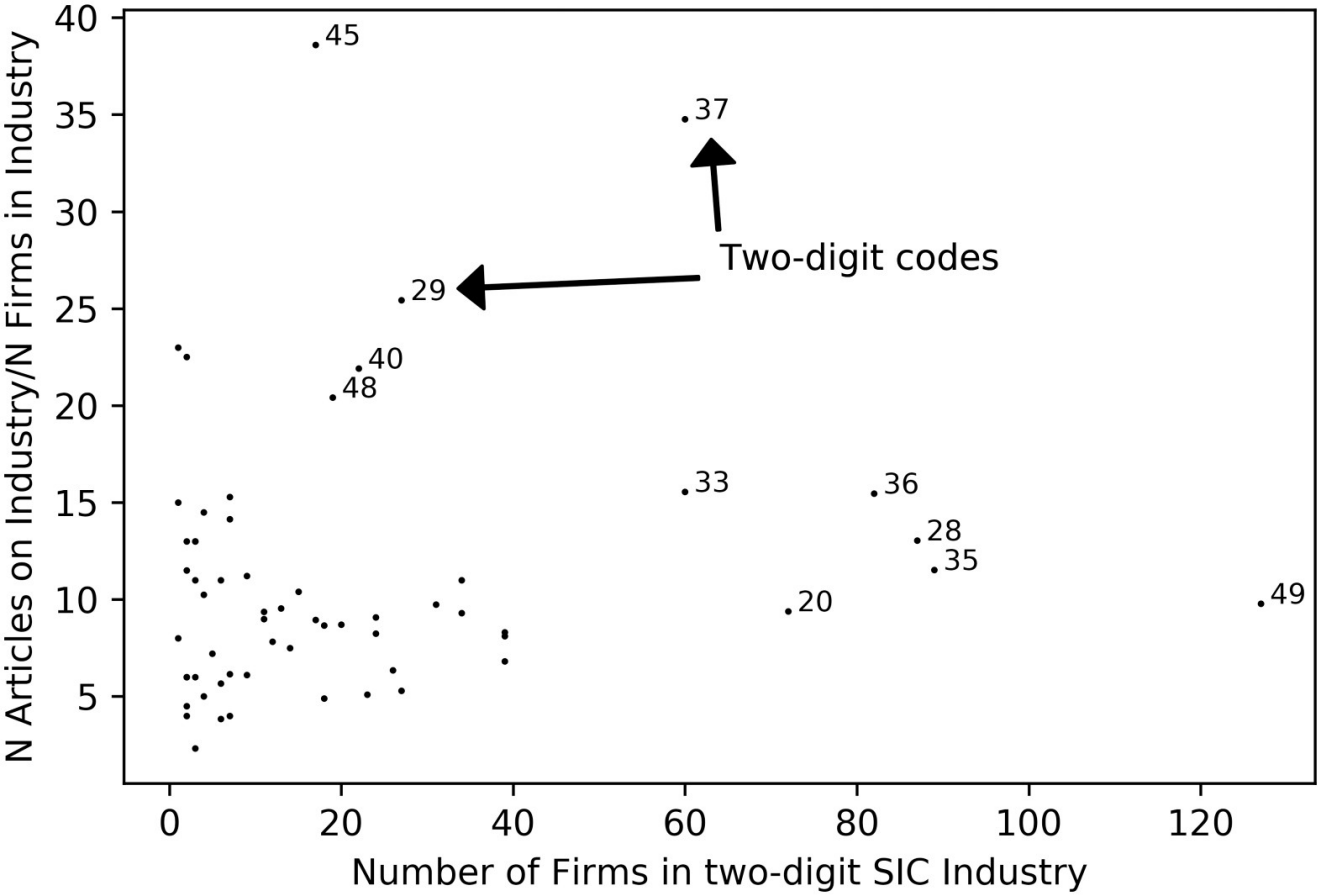
Number of firms in two-digit SIC industry. Number of firms in two-digit SIC industry are plotted against the number articles published in WSJ divided by number of firms in the industry.

Of the 1,265 stocks in the sample, WSJ published at least one article on 1,237 or over 98% of the stocks. This is in contrast to later years when Peress [7] finds that only 59% of NYSE-listed firms are covered by the WSJ during his sample years. The average firm is covered 12 times/year but the number of articles per firm varies widely and is highly skewed toward some firms receiving a lot of coverage. In contrast, the 61 firm-specific articles that are published in WSJ per day is consistent over time with a low variance. From CRSP, I am also able to identify the primary SIC code of every firm at that time. To summarize WSJ coverage across industries I group firms by their two-digit SIC code. There are several two-digit SIC code industries that lack any presence of NYSE-listed firms. But, focusing on the 60 SIC codes that are represented in the sample, there is substantial variance in the number of firms in industries. Obviously, WSJ’s coverage is likely to be skewed toward industries with more firms. Thus, to understand whether WSJ pays more attention to certain industries, I normalize the number of articles published on firms in each industry by the number of firms in that industry. While summary statistics on the normalized coverage on each industry appear in the table, Fig 2—in which I plot the number of firms in the industry versus the number of WSJ articles on that industry divided by number of firms in the industry—provides a better picture of the important details.

It is clear that some industries receive substantially more coverage in WSJ even after adjusting for the number of firms in that industry while others receive substantially less coverage than warranted. In particular, there are five 2-digit SIC code industries that receive more coverage: 45 (airlines and airfreight carriers), 37 (the manufacturing of aircraft, autos, boats, trucks, and parts for all of these), 29 (oil exploration and refining), 40 (railroad), and 48 (radio and television broadcasting and telephone and telegraph communications). All of these industries are engaged in the transportation industry whether it is the transportation of goods or people or the manufacturing of vehicles and parts or providing energy for these vehicles or transporting electronic signals (broadcasting and telecommunications). Likewise, there are five 2-digit industries that received less coverage than warranted: 49 (utilities and sanitary services), 35 (engines, turbines, farm and industrial machinery), 28 (chemicals and allied products like pharmaceuticals), 36 (electronic and electrical equipment except computers) and 20 (food and kindred products). There could be a number of reasons why these biases in coverage are present. For instance, they could be related to the tastes and preferences of WSJ’s subscriber base, or the expertise of WJS’s staff, or due to an abundance of newsworthry events in 1970 in the industries that are covered more frequently. From the available data, I am unable to identify which of these explanations are more likely.

Although NYSE-listed firms are generally thought to be large, since there was no NASDAQ at that time, the range of size is vast with the smallest firm having a market value of equity of only $7 million and the largest one worth well over $40 billion. The mean and median daily turnover in shares is close to one-tenth of 1%. The lowest is approximately half of that, while the maximum is twice that. Thus, daily turover fluctuates in a fairly narrow band despite the vast range in the size of firms. The average distance of NYSE-firm headquarters is approximately 1,000 miles. That statistic is somewhat misleading because firm headquarters appear to cluster in large metropolitan areas. Approximately 21% of the firms are headquartered within 10 miles of WSJ’s headquarters (the NY-NJ-CT metropolitan area) with additional large clusters in Chicago and Los Angeles and smaller ones in other metropolitan areas.

Before proceeding to the main results of the paper, it will be useful to get some insights into which firms appear more frequently in the WSJ after removing the industry effects documented previously. In Table 2, I present the results from a Poisson regression with a negative binomial model form with number of WSJ articles as the dependent variable and size of firm (log market value of equity at the end of 1969 or on the first trading day in 1970 whichever is earlier), firm mean turnover as independent variables. In the second regression, the distance of firm’s headquarters from WSJ’s headquarters appears as an additional independent variable. Both regressions contain the mean per firm number of articles for two-digit SIC of the industry as a control variable. The coefficients on this control variable are not reported but are always statistically significant. Once the propensity of the firm’s industry to appear in WSJ has been controlled for in this way, it is clear that larger firms are much more likely to receive coverage; additionally, firms with higher turnover are also more likely to appear in WSJ. This is a new finding and lends some support to the idea that WSJ provides greater coverage of firms that investors are more interested in. Finally, how far a US-based firm is located from WSJ headquarters has virtually no impact on whether the firm will appear in the WSJ or not.

**Table 2:** Which firms does WSJ write about? Coefficients and *z*-statistics (in parentheses) from a Poisson regression with a negative binomial model form with the number of WSJ articles published about each firm as the dependent variable and log of market value of equity (at end of December 1969 or first day of trading, whichever is later), mean daily stock turnover of each firm, distance of firm headquarters from WSJ headquarters and the number of WSJ articles on 2-digit SIC industry/number of firms in industry (coefficients not reported but available from the author) as independent variables.

Intercept	log(MVE)	Mean turnover	Distance	N
-0.61***	0.44***	95.71***		1,265
(-7.12)	(29.37)	(7.43)		
-0.58***	0.44***	99.10***	-0.000025	1,234
(-6.52)	(29.20)	(7.66)	(-1.62)	

*Significant at the 10% level.

**Significant at the 5% level.

***Significant at the 1% level.

## Results

The main results of this study—based on panel regressions—appear in Table 3. With 1,265 stocks and 254 days of trading, the sample size could theoretically be as large as 321,310 observations. However, not every stock existed on every day and occassionally volume and return are found to be missing. I lose fewer than 2,000 observations (<1% of the sample) in the panel regressions, but the number varies depending on the variables that appear in each regression. The exact number of observations appear in each of the columns. The first three columns are devoted to turnover, the fourth to dispersion, and the last to return. All regressions reported in the table contain dummy variables to control for day-of-the-week effects and for each stock and the standard errors for the test statistics are clustered by firm.

**Table 3:** Effect of strike on turnover, dispersion, and returns. Coefficients and *t*-statistics (in parentheses) from an OLS regression with daily turnover, dispersion, and returns as the dependent variables and a dummy variable that indicates whether WSJ wrote about that firm on that day (Article dummy), the positve (RESID+) and negative (RESID-) residuals from a market-model regression, a dummy variable for when the residual was positive (RESID+ dummy), day-of-the-strike dummies (SD -1, SD1, SD2, …), and interaction terms as independent variables. Dummy variables for day-of-the-week and for each firm are also included. Standard errors are clustered by firm.

	Turnover 1	Turnover 2	Turnover 3	Dispersion	Returns
Intercept	0.00039***	0.00039***	0.00039***	0.0259***	0.00033***
	(14.97)	(15.07)	(15.09)	(74.43)	(10.11)
Article dummy	0.00028***	0.00028***	0.00028***	0.0039***	-0.0013***
	(5.59)	(5.56)	(5.55)	(6.45)	
RESID+ dummy	0.00009***	0.00009***	0.00009***		
	(3.77)	(3.81)	(3.80)		
RESID+	0.0341***	0.0341***	0.0341***		
	(14.05)	(13.96)	(13.98)		
RESID-	-0.0412***	-0.0412***	-0.0412***		
	(-13.60)	(-13.52)	(-13.54)		
SD -1		0.00006		0.0136***	0.0157***
		(0.74)		(18.42)	(16.47)
SD 1		-0.00032***	-0.00032***	0.0008	0.0017**
		(-7.43)	(-7.39)	(1.51)	(2.25)
SD 2		-0.0002***	-0.0002**	0.0047***	-0.018***
		(-2.65)	(-2.56)	(7.32)	(-23.24)
SD 3		-0.0001	-0.0001**	0.0099***	-0.0278***
		(-1.44)	(-2.21)	(12.99)	(-31.00)
SD 4		0.0002***	0.0002***	0.0200***	-0.0234***
		(3.20)	(3.34)	(16.59)	(-23.67)
SD 5		-0.0001	-0.0001	0.0153***	-0.0072***
		(-1.06)	(-0.96)	(16.28)	(-7.94)
SD 6		-0.0002***	-0.0002***	0.028***	-0.04114***
		(-3.55)	(-4.51)	(22.98)	(-35.02)
SD 7		0.0001	0.0001	0.0273***	-0.0151***
		(1.16)	(1.21)	(23.60)	(-12.83)
SD 8		0.0003***	0.0003***	0.0271***	0.033***
		(4.37)	(3.16)	(30.98)	(26.51)
SD AFTER		0.00002		0.0229***	0.0266***
		(0.18)		(24.93)	(23.64)
SD 1 * Article dummy			0.00	0.0100	-0.0017
			(0.00)	(1.75)	(-0.33)
SD 2 * Article dummy			-0.00003	-0.0032	0.0015
			(-0.13)	(-0.75)	(0.37)
SD 3 * Article dummy			0.0008	0.0062	-0.0102**
			(0.91)	(1.43)	(-2.11)
SD 4 * Article dummy			-0.0003	-0.0025	-0.0003
			(-1.50)	(-0.59)	(-0.06)
SD 5 * Article dummy			-0.0001	-0.0001	0.0029
			(-0.49)	(-0.02)	(0.59)
SD 6 * Article dummy			0.0012	0.0035	-0.0073
			(1.33)	(0.51)	(-1.16)
SD 7 * Article dummy			-0.0002	0.0037	0.0011
			(-0.59)	(0.68)	(0.18)
SD 8 * Article dummy			0.0006	0.0032	-0.0002
			(1.21)	(0.51)	(-0.05)
Adjusted *R*^2^	0.067	0.067	0.067	0.025	0.03
*F*	56.68***	27.99***	23.06***	109.88***	205.65***
N	319,396	319,396	319,396	319,541	319,524

*Significant at the 10% level.

**Significant at the 5% level.

***Significant at the 1% level.

In the Turnover 1 regression, I model the relation between turnover and potential explanatory variables without regard to whether there was a strike or not. Generally speaking, trading is motivated by either noise traders or by informed traders. To understand the effect of information on trading, I include a dummy variable, “Article dummy” that indicates whether an article on that stock was published or not and a firm-specific residual from a market-model regression of that firm’s returns against the CRSP value-weighted index. The residual captures the positive or negative tenor of news specific to every firm. To distinguish between the possibility that good and bad news may affect trading in different ways, I regress a positive residual (“RESID+”) separately from a negative residual (“RESID-”). Further, I also include a dummy variable (“RESID+ dummy”) when the residual is positive to allow the intercept of the relation between turnover and residual to be different depending on the tenor of the news.

I find a positive and highly significant relation between daily turnover and whether WSJ published an article on that stock. In fact, comparing the coefficient on the article dummy to the average daily turnover it is clear that turnover rises by more than 20% when WSJ publishes an article on a firm. This increase in turnover puts trading for that day in the top quartile of turnover days. A mundane day of trading is thus transformed into an active day of trading.

When the firm “beats the market” the average turnover is higher as the coefficient on RESID+ is positive and highly significant. Further, turnover responds more strongly to a negative residual than to a positive residual which is consistent with the Chordia et al. [29] finding that aggregate trading activity increases more in a down market than in an up market. Both effects are economically and statistically highly significant. In regressions not displayed in the table (but available from me), I also study the effect of firm size on turnover by creating dummy variables for each firm based on the size quintile each firm falls in. Once dummy variables for each firm are replaced by size-quintile dummies, there are statistically significant differences in turnover by size quintile but the pattern is non-monotonic, thus making it difficult to attach any meaning to the effect of firm size on turnover.

In Turnover 2, the panel now contains dummy variables for each day of the strike (“SD 1”, “SD 2”, etc.). Additionally, to ensure that any significant results are related to the occurrence of the strike, I also add dummy variables for the day before the beginning of the strike and the day after the last day of the strike (“SD -1” and “SD AFTER”). The coefficients on the day before the strike and the first day after the strike are both statistically insignificant, suggesting that nothing unusual was occurring to turnover around the days of the strike. On the first day of the strike, turnover falls by a statistically and economically significant 32%; on the second day turnover falls by a statistically and economically significant 20%. on the third day there appears to be a return to a normal turnover as the coefficient is statistically insignificant. By the fourth day, turnover has actually risen to 20% above normal. For the rest of the days, turnover appears to fluctuate below and above normal with one normal day of trading thrown in. In results not displayed in any table, I also calculate the percentage change in total dollar-trading volume (price × volume) for every day of the year. The largest one-day fall in dollar-trading volume (-45%) occurrs on the first day of the strike.

Thus, the evidence is unequivocal that the strike by the Newspaper and Mail Deliverers’ Union led to a significant reduction in trading on the first few days of the strike. This is all the more significant in light of the fact that only 100,000 copies out of WSJ’s total circulation of over 1.2 million were not delivered and there was no disruption in Dow Jones News Service. As the strike progressed, however, turnover gravitated to a normal level, then above normal levels, followed by uneven changes. That there is anecdotal evidence suggesting that consumers of media substitute another source of media for their preferred source when their preferred is unavailable may explain the turnover reverting to normal levels as the strike progressed. This evidence provides a more nuanced view of clogging of information and its effect on the market in a dynamic world where seekers of information react to unblock their access to it or substitute other sources. Below, I will provide some potential explanations for why turnover may have risen to an above-normal level.

It is important to understand the cross-sectional determinants of the variance in turnover during the strike days. In Turnover 3, I display the coefficients on a regression that includes, in addition to the variables in Turnover 2, interaction terms between each strike day and the “Article dummy”. Although the WSJ was not delivered in the NY-metropolitan area, over 90% of its subscribers did receive their copies. Thus, it is still possible that news published in WSJ led to trading in the firms that were written about. However, none of the strike and article interaction dummy terms are statistically significant.

In regressions not reported in the paper but available from me, I replace the firm dummies with size-quintile dummies and also let each strike-day dummy interact with the size-quintile dummies. The interaction terms are all insignificant, so the change in turnover during the strike is unrelated to the size of the firms. This is in contrast to Peress [7] who finds that the fall in turnover is confined to the bottom three size quintiles in his sample. Together with the 45% one-day drop in dollar-trading volume, my results show that information clog affects the market as a whole without regard to the size of the firm. I also divide my sample of firms into distance quintiles and find that the fall in turnover during the strike is unrelated to how far the firm is from WSJ. Finally, I also let the strike dummies interact with the 2-digit SIC dummies. As shown previously, certain 2-digit SIC industries receive more than their “fair” share of coverage in WSJ. Firms in such industries may bear the brunt of the fall in turnover due to clogging of information during the strike. However, I find no evidence to support this idea.

In the remaining regressions in the table, I examine the effect of the strike on daily dispersion and daily return. If dispersion is related to the flow of good and bad news and that flow gets clogged for any reason, we should expect to find lower dispersion during the strike. Because dispersion and returns are both correlated with residuals, I leave residuals out of these regressions.

On the first day of the strike dispersion is unchanged. For the remaining days, dispersion is statistically higher than on non-strike days. However, dispersion is statistically significantly higher even on the day before the strike began and the day after the strike ended. Thus, I’m unable to rule out the possibility that the higher dispersion during the strike is actually due to a period in the market when dispersion was higher than normal. At first blush, the evidence on dispersion is inconsistent with Peress’ finding that it falls in his sample. However, when viewed in the larger context of a day before and after the strike, my findings on dispersion may be unrelated to the strike—rather, just part of a period when dispersion was swollen.

On virtually all strike days except the first and the last, returns are a statistically significantly negative. The “Article dummy” on returns is also statistically significantly negative, which suggests that the news carried by WSJ specific to firms during the year was negative. On strike days, however, there is no particular reason why news should have been significantly negative. This strike was confined to the previously-mentioned grievance that the Union’s members had with WSJ and was completely unrelated to other unions’ activities during that time with no implications for the overall economy.

Finally, I turn my attention to return comovement between stocks before, during, and after the strike. For each stock in the sample, I calculate the Pearson product-moment correlation coefficient of returns for that stock and the CRSP value-weighted index for the eight days of the strike, then average those correlation coefficients over all stocks. Similarly, for the eight trading days immediately before and after the strike, I calculate a pre-strike and a post-strike average correlation coefficient. The average pre-strike correlation coefficient is 0.478; during the strike it is 0.657, and after the strike it is 0.503. Using a Fisher transformation, I find that the *p*-value for the null hypothesis that the pre-strike correlation is less than the during-strike correlation is <0.0001. Further, the *p*-value for the null hypothesis that the post-strike correlation is less than the during-strike correlation is also <0.0001. It is clear that return comovement increases substantially during the strike period. This evidence can be intrepreted as a fall in market efficiency due to the clogging of information during the strike as economy-wide and industry-wide information continues to be impounded into prices, but not as much firm-specific information is impounded into prices leading to greater return comovement.

## Conclusion

I study the 1970 Newspaper and Mail Deliverers’ Union strike against WSJ and its effect on the stock market. A natural question that arises, of course, is how this research is relevant to the substantially changed world that we live in today as well as the changes that will occur in the future? Consumers of news now receive it from many more sources than they did previously. However, few of these sources subscribe to the same journalistic standards that the best sources do. Further, it is clear that many of these sources can be manipulated in a way that is not transparent to the ultimate consumers. Even a casual examination of WSJ’s history [10] suggests that the founders of Dow Jones & Co., WSJ’s parent, built the firm on a reputation of truth, objectivity, relevance, and timeliness. Thus, it is vital to understand the impact of the clogging of important sources of news on real events *particularly* in the context of the massive transformation that media and news has gone through and will continue to in the future.

I find that turnover and dollar-trading volume on the NYSE fall substantially and statistically significantly during the early days of the strike, then level off and even rise toward the end of the strike. The fall as well as the subsequent increase occurs across all size quintiles of NYSE firms. Previous research on the effect of newspaper strikes on the stock market has found that smaller firms are more affected than larger firms, but that is not the case in the US. Further, the fall in turnover is unaffected by how far the firm is from WSJ’s headquarters. My findings present a more nuanced view of how an exogenous clogging of news or information affects the market and are consistent with the idea that news-deprived consumers seek out a substitute when their preferred source is unavailable for some reason.

The evidence also further supports the idea that the leading sources of news have a substantial impact on the financial market—the publication of an article in WSJ transforms a mundane day of trading in a particular stock to an active day of trading. Further, trading increases in response to unusually good or bad news but the effect is larger for bad news. Overall, all of this evidence is further confirmation of the idea that there exists a causal relation between media coverage and trading in the financial market.

I also provide evidence that return comovement increases during the strike. Return comovement is a result of a decrease in market efficiency. Thus, this is the first evidence that I am aware of that confirms the idea that that the clogging of news reduces market efficiency. It appears that economy-wide and industry-wide news continues to be incorporated into prices, but the incorporation of detailed firm-specific news—the *raison d’etre* of specialized outlets like the WSJ—into prices is reduced.

Further, although I find that dispersion in price rises during the strike this increase appears to be related to an unusual increase in dispersion in the period surrounding the strike. Thus, I am unable to attribute the increase in dispersion solely to the occurrence of the strike. I also find that returns are negative throughout the strike. Additionally, I find that the general tenor of the news was also negative in 1970. Both of these are most likely just random artifacts, unrelated to the context in which these events occurred.

It is clear that WSJ publishes more news on larger firms and firms that operate in certain industries. However, after controlling for these effects, I discover that it publishes more on firms that are more actively traded. In that sense, WSJ is geared toward its customers.

Overall, it is clear that traditional outlets such as newspapers are an important source of reliable information. While the present conventional wisdom suggests that journalism has a smaller role to play in the technologically changed world that we live in today, reliable, truthful, and objective sources of news will continue to play an important role. Further, when the normal flow of information is interrupted due to geo-political events such as wars or pandemics, governments are well-advised to pay close attention to unclogging any sources of clogged information.
